# Applications and challenges of photodynamic therapy in the treatment of skin malignancies

**DOI:** 10.3389/fphar.2024.1476228

**Published:** 2024-09-19

**Authors:** Yunqi Hua, Xiaoling Tian, Xinyi Zhang, Ge Song, Yubo Liu, Ye Zhao, Yuqian Gao, Fangrui Yin

**Affiliations:** ^1^ Department of Medical Oncology, Baotou Cancer Hospital, Baotou, China; ^2^ Department of Graduate School, Baotou Medical College, Inner Mongolia University of Science and Technology, Baotou, China; ^3^ Department of Public Health, International College, Krirk University, Bangkok, Thailand; ^4^ Department of Rheumatology, The First Affiliated Hospital of Baotou Medical College, Baotou, China

**Keywords:** photodynamic therapy, skin malignant tumors, therapeutic effect, limitation, treatment

## Abstract

Photodynamic Therapy (PDT), as a minimally invasive treatment method, has demonstrated its distinct advantages in the management of skin malignant tumors. This article examines the current application status of PDT, assesses its successful cases and challenges in clinical treatment, and anticipates its future development trends. PDT utilizes photosensitizers to interact with light of specific wavelengths to generate reactive oxygen species that selectively eradicate cancer cells. Despite PDT’s exceptional performance in enhancing patients’ quality of life and prognosis, the limitation of treatment depth and the side effects of photosensitizers remain unresolved issues. With the advancement of novel photosensitizers and innovative treatment technology, the application prospects of PDT are increasingly expansive. This article delves into the mechanism of PDT, its application in various skin malignancies, its advantages and limitations, and envisions its future development. We believe that through continuous technological enhancements and integration with other treatment technologies, PDT has the potential to assume a more pivotal role in the treatment of skin malignancies.

## 1 Introduction

Skin malignant tumors, which encompass basal cell carcinoma, squamous cell carcinoma, and malignant melanoma, represent a common type of cancer globally. Over recent decades, the incidence of these tumors has risen, presenting a considerable challenge to public health (Di Bartolomeo et al., 2022). Although traditional treatments such as surgery, radiation therapy, and chemotherapy can help manage the progression of the disease to some degree, they come with notable limitations, including physical trauma and severe side effects. Consequently, there is an increasing emphasis on finding more effective and less harmful treatment alternatives.

**FIGURE 1:**
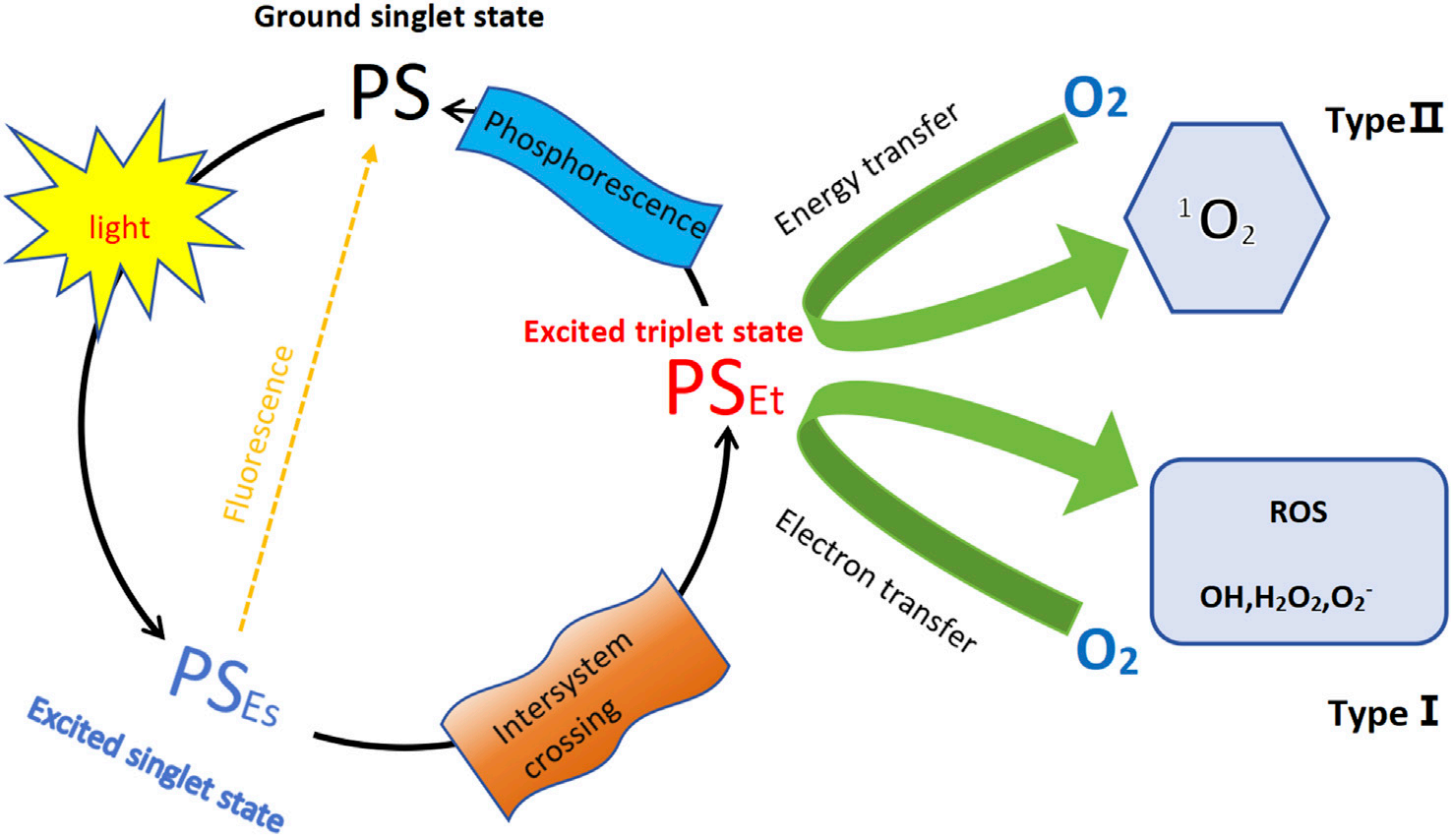
Schemat ic illustration of photodynamic therapy (PDT). The diagram illustrates the PDT mechanism involving a PS. The process starts with the PS in its ground singlet state. Upon absorption of light, the PS transitions to its excited singlet state. From here, it can undergo one of two primary pathways (Di Bartolomeo et al., 2022): fluorescence–the PS can release energy and return to the ground state, emitting fluorescence (Akhtar et al., 2024); inter-system crossing–the PS transitions to a triplet state. In the triplet state, the PS can follow two distinct paths: Type Ⅰ reaction–the PS transfers an electron to molecular oxygen, generating other ROS, including superoxide (O_2_^−^), hydrogen peroxide (H_2_O_2_) and hydroxyl radicals (OH); Type Ⅱ reaction–the PS transfers energy to molecular oxygen (O_2_), producing singlet oxygen (^1^O_2_); Additionally, from the triplet state, the PS can relax back to the ground state, emitting phosphorescence.

Photodynamic therapy (PDT) is a non-invasive and effective treatment for cancer that utilizes specific wavelengths of light to activate photosensitizers (PS) in an oxygen-rich environment. When the PS is excited by light, it transitions from an excited singlet state to a long-lived triplet state, subsequently reacting with ground state (triplet) O_2_ to generate reactive oxygen species, including singlet oxygen and free radicals (Akhtar et al., 2024). This mechanism is illustrated in Figure 1. PDT has gained prominence as a significant method for treating skin malignancies due to its advantages of being minimally invasive, targeted, and repeatable (Wang et al., 2021). The fundamental principle behind PDT is that the photosensitizer, upon exposure to specific light wavelengths, produces singlet oxygen and other reactive oxygen species, which damage the cell membranes and organelles of cancer cells, ultimately leading to apoptosis or necrosis (Varzandeh et al., 2021). In contrast to traditional chemotherapy and radiotherapy, which rely on toxic chemicals and ionizing radiation, PDT is considered a relatively safe, FDA-approved anticancer intervention (Modi et al., 2024).

PDT has shown significant effectiveness in the treatment of skin cancers, particularly in the elimination of basal cell carcinoma and squamous cell carcinoma, while also contributing to lower recurrence rates (Szeimies and Karrer, 2021). Furthermore, PDT has proven beneficial in addressing other skin lesions such as actinic keratosis and Bowen’s disease (Kochergin et al., 2024). Nevertheless, despite these advantages, PDT encounters several challenges, including the selection of suitable photosensitizers, limitations in light penetration depth, and the possibility of side effects (Kawczyk-Krupka et al., 2020).

In summary, PDT shows significant clinical benefits and offers a wide range of potential applications for the treatment of skin malignancies. This article will explore several important aspects of PDT, including its mechanisms of action, underlying principles, various applications, advantages and limitations, recent research developments, and future trends. By examining these elements, we aim to provide valuable insights that can enhance strategies for effectively treating skin malignancies.

## 2 The mechanism and principle of photodynamic therapy

The photodynamic reaction encompasses several key biological processes, including the absorption, distribution, and photoexcitation of the photosensitizer, along with the generation of reactive oxygen species (ROS). The photosensitizer can be introduced into the body through various methods such as injection, topical application, or oral administration, and it tends to selectively accumulate in tumor tissue. When specific wavelengths of light are directed at the tumor, they activate the photosensitizer, prompting it to produce ROS (Hsia et al., 2023). These ROS inflict damage on the membranes, proteins, and nucleic acids of tumor cells, leading to oxidative stress. This process ultimately results in cell death through apoptosis or necrosis and also triggers an immune response that aids in the further elimination of tumor cells (Mo et al., 2021; Nowak-Perlak et al., 2023).

### 2.1 Mechanism of action of photosensitizers

PDT is a treatment that relies on the interaction between photosensitizers, specific light wavelengths, and oxygen (Luo et al., 2024). It utilizes particular light wavelengths to activate photosensitizers, which are compounds employed in cancer treatment and as imaging agents to help locate tumors. Both the FDA and EMA have approved first-generation and second-generation photosensitizers for clinical use across various stages of cancer. Currently, researchers are working on developing third-generation photosensitizers aimed at enhancing specificity for cancer cells and increasing their accumulation in tumors (Modi et al., 2024) (Table1). During PDT, photosensitizers are excited by light of specific wavelengths, leading to type I and type II reactions that produce ROS. These ROS inflict oxidative damage on cellular components, ultimately resulting in cell death. This approach selectively targets and destroys diseased cells while minimizing damage to healthy tissue (Akhtar et al., 2024).

**TABLE 1:** Overview of various generations of photosensitizers.

	Example	Peculiarity
First generation	Hematoporphyrin Derivative (HpD)	These mixtures exhibit weak light absorption and minimal tissue penetration, leading to their accumulation in normal tissues and skin, which can cause significant phototoxicity
Second generation	5- Aminolevulinic Acid (5-ALA), Benzoporphyrin (BPD),and Porphyrin Tin	These photosensitizers exhibit enhanced light absorption, deeper tissue penetration, and shorter photosensitivity periods, thus reducing the risk of sensitivity after treatment
Third generation	amino acid-conjugated photosensitizers, polymer-conjugated photosensitizers, and protein or sugar-conjugated photosensitizers, and others are still in the research stage	Most third-generation photosensitizers are improved versions of second-generation compounds that use biological agents or recognition chemicals to enhance lesion targeting. In addition to these, novel photosensitizers include oxygen-independent types that oxidize water to produce hydroxyl radicals for tumor cell destruction, as well as those enhanced with nanotechnology for improved tumor targeting. Additionally, some of these novel photosensitizers can effectively perform photodynamic therapy in low-oxygen environments

### 2.2 The selection of photosensitizers and the impact of wavelength on treatment efficacy

The choice of PS and the specific wavelengths used are crucial factors that significantly influence the effectiveness of PDT. The properties of the photosensitizer, along with the components involved in PDT, play a vital role in determining the success of the treatment. Commonly utilized photosensitizers include porphyrins, phthalocyanines, and ruthenium complexes (Jiang et al., 2022). An ideal photosensitizer should exhibit a high light absorption coefficient, particularly in the longer wavelength range, as longer wavelengths facilitate deeper tissue penetration (Kessel, 2023). For optimal penetration depth, the photosensitizer should absorb light within the wavelength range of 650–900 nm, while also exhibiting low dark toxicity to reduce adverse effects on surrounding healthy tissues (Hu et al., 2015; Sun et al., 2024). Additionally, the intensity and duration of light exposure are critical factors that can impact treatment outcomes; excessive light intensity may cause damage to normal tissues, whereas insufficient intensity might not adequately activate the photosensitizer (Verebová et al., 2020).

## 3 Application of photodynamic therapy in malignant skin tumors

Skin cancer, the most common form of cancer, is increasingly prevalent and is classified into two main categories: melanoma and non-melanoma skin cancer (NMSC) (Jones et al., 2020). NMSC arises from non-melanocyte cells and primarily includes basal cell carcinoma and squamous cell carcinoma, which originate from keratinocytes (Karimkhani et al., 2015). On the other hand, melanoma results from mutations in melanocytes, often linked to sun exposure during adolescence, especially from sunburns experienced between the ages of 15 and 20.

### 3.1 Non-melanoma skin cancer (NMSCs)

The primary risk factor for non-melanoma skin cancer is exposure to ultraviolet (UV) radiation, which can cause DNA damage (Nehal and Bichakjian, 2018). Surgical excision or electrodessication is commonly recommended as treatment options; however, these procedures may lead to pain and scarring, particularly in sensitive areas of the body (Yang et al., 2024). Therefore, it is essential to find a balance between the effectiveness of the treatment and the patient’s quality of life. Aminolevulinic acid photodynamic therapy (ALA-PDT) has shown effectiveness in treating skin diseases and can enhance patient outcomes when utilized before surgery (Eisen et al., 2021). In cases where surgery or radiotherapy is not feasible, neoadjuvant therapy becomes necessary. PDT is recognized as a non-invasive and safe treatment alternative for non-melanoma skin cancers (Yan et al., 2024).

#### 3.1.1 Basal cell carcinoma (BCC)

Basal cell carcinoma (BCC) is the most prevalent type of non-melanoma skin cancers (NMSCs) and is categorized into nodular, superficial, and infiltrative types. Surgical excision is an effective treatment for primary BCC, achieving a 5-year recurrence rate between 2% and 8%. In cases involving high-risk or recurrent patients, surgery may serve as a palliative option, which can be complemented by neoadjuvant therapy (Espiñeira Sicre et al., 2023). PDT has emerged as a neoadjuvant treatment for BCC, aimed at reducing tumor burden and decreasing local incidence (Zelin et al., 2021; Sato-Akushichi et al., 2022). Aminolevulinic acid (ALA)-based PDT is frequently utilized for various tumors, including bladder cancer, basal cell carcinoma, and head and neck cancer, and has also proven effective for skin conditions such as actinic keratosis, psoriasis, acne, and Bowen’s disease (Lou et al., 2023). For instance, Liao C and colleagues successfully treated a 91-year-old patient with locally advanced BCC using a combination of modified ALA-PDT, holmium yttrium aluminum garnet laser, and HiPorfin-PDT. Following six sessions of modified ALA-PDT, the tumor size significantly reduced from 15 mm to 4 mm, demonstrating that neoadjuvant PDT can avoid the need for skin grafting and preserve normal hair follicles (Liao et al., 2021a). This approach offers distinct advantages in managing multiple lesions, especially those located on the face and scalp (Liao et al., 2021b).

Espiñeira Sicre et al. (2023) presented a case involving recurrent multifocal BCC that did not respond to conventional treatments following radiotherapy. As an alternative approach, they proposed neoadjuvant PDT, which proved effective in significantly reducing tumor volume and eliminating adjacent lesions. In cases of giant basal cell carcinoma, traditional surgical excision can be quite invasive, making neoadjuvant PDT a valuable non-invasive option (Mikhaimer et al., 2010). Similarly, Madan V et al. documented three patients with giant BCC who underwent treatment with PDT combined with topical imiquimod. This combined treatment resulted in lesion size reductions of 40%, 25%, and 22%, facilitating minimal surgical excision and consequently lowering postoperative complications (Tierney et al., 2011).

Neoadjuvant PDT has been shown to effectively decrease the size of lesions, reduce the extent of surgical trauma, and yield positive cosmetic outcomes in the management of BCC.

#### 3.1.2 Squamous cell Carcinoma (SCC)

Cutaneous squamous cell carcinoma (cSCC) is a prevalent form of skin cancer associated with factors such as UV exposure, chemical exposure, genetic predispositions, and the use of immunosuppressive medications (Chen et al., 2024; Fang et al., 2022). Neoadjuvant PDT has been shown to effectively reduce cSCC lesions; however, treating lesions on the lips presents unique challenges, often necessitating surgical intervention or radiotherapy (Jeremic et al., 2011; Li X. et al., 2023). ALA-PDT emerges as a promising alternative for achieving complete remission in these cases. Research indicates that a combination of superficial excision followed by local adjuvant PDT can yield positive outcomes for cSCC located on the lips (Yan et al., 2020). While traditional ALA-PDT can result in considerable pain, which may hinder patient tolerance, modified PDT has demonstrated improved effectiveness and better tolerance among patients (Zeng et al., 2022).

Enhanced 5-aminolevulinic acid photodynamic therapy (M-PDT) represents a promising new strategy for the treatment of cSCC, demonstrating both effectiveness and good tolerability in patients. The pioneering work by Chen et al. (2024) revealed that M-PDT triggers pyroptosis, a form of programmed cell death, in cSCC cells through the ROS-JNK-NLRP3 signaling pathway. Their findings indicate that the use of inhibitors targeting NLRP3, JNK, and ROS can significantly diminish pyroptosis, as well as the release of key inflammatory markers such as N-GSDMD, cleaved caspase-1, and mature interleukin 1 Beta(IL-1B). On the other hand, activating JNK can enhance the assembly of the NLRP3 inflammasome, thereby promoting pyroptosis. This research elucidates the underlying molecular mechanisms by which M-PDT operates in the treatment of cSCC, providing a valuable theoretical framework for its clinical application.

Research conducted by Zeng et al. (2023) demonstrates that M-PDT effectively inactivates the ROS-mediated Akt/mTOR pathway. This inactivation plays a crucial role in preventing the fusion of autophagosomes with lysosomes, which subsequently disrupts the autophagic flow in cSCC cells. As a result, there is an accumulation of autophagosomes and a decrease in the activity of cSCC cells. The findings from this study suggest that M-PDT holds significant promise as a treatment for cSCC, especially when autophagy is inhibited to enhance its therapeutic efficacy.

Studies and case reports indicate that the combination of neoadjuvant PDT with surgery or laser treatment is effective for NMSCs, leading to a low recurrence rate and good patient tolerance. Neoadjuvant PDT not only reduces the risk of recurrence but also helps prevent the emergence of new NMSCs, which can occur due to field cancerization. It is advisable to adopt a multidisciplinary team approach to manage patients undergoing neoadjuvant PDT effectively. In summary, neoadjuvant PDT presents a promising treatment option for NMSCs, especially in cases where surgery may not be the preferred method. Additionally, this therapy can significantly lower the likelihood of recurrence and inhibit the development of new NMSCs arising from areas with pre-cancerous changes.

### 3.2 Melanoma

Melanoma is a severe form of skin cancer that arises from melanocytes and is characterized by its complex heterogeneity (Fadadu and Wei, 2022). The metastatic variant of melanoma is particularly concerning due to its high mortality and recurrence rates, underscoring the urgent need for new treatment options, despite the advancements made in immunotherapy (Volety et al., 2024). One promising approach is PDT, a minimally invasive treatment that effectively targets and destroys tumors, and is increasingly being explored in the context of melanoma treatment (Naidoo et al., 2018). A more innovative strategy is PIT, which combines the tumor-destroying effects of PDT with enhanced anti-tumor immune responses (Mohiuddin et al., 2023). Unlike traditional PDT, which mainly focuses on reducing tumor size, PIT aims to boost the immune system’s ability to recognize and attack cancer cells. This method not only increases the immunogenicity of tumors but also diminishes the presence of regulatory immune cells that can lead to resistance against immunotherapy (Lobo et al., 2023; Kato et al., 2022).

PIT specifically targets localized tumors, which helps to minimize off-target toxicity and adverse effects, making it a promising alternative to chemotherapy for melanoma (Pham et al., 2023). By inducing systemic immune responses directly at the tumor site, PIT enhances its effectiveness when used in combination therapies, potentially benefiting other types of cancer as well (Nkune and Abrahamse, 2024). Additionally, combining PIT with immune checkpoint blockade or adoptive cell therapy may elicit a strong systemic anti-tumor immune response.

L.W.'s *in vivo* experiments revealed that Chlorin e6-C-15-ethyl ester (LS-HB) effectively targets and kills malignant melanoma cells, specifically B16F10 and A375, when exposed to 660 nm light. The primary mechanism of cell death at lower doses is through the induction of apoptosis, which operates via the mitochondrial caspase-9/caspase-3/PARP pathway. In contrast, at higher doses of 8 μg/mL, the treatment results in cell necrosis. Additionally, LS-HB PDT demonstrated significant anti-tumor effects *in vivo*, likely due to the damage inflicted not only on the tumor cells but also on the blood vessels within the tumor tissues. As a result, LS-HB stands out as a promising photosensitizer for cancer treatment (Wang et al., 2022).

Overall, the application of PDT in skin malignancies has achieved significant clinical efficacy, and its combination with other treatment methods can further enhance therapeutic effects and patient prognosis. In the future, with the development of new photosensitizers and therapeutic techniques, the application prospects of PDT in the treatment of skin malignancies will be even broader.

## 4 Advantages and limitations of photodynamic therapy

PDT presents several advantages compared to traditional cancer treatments, particularly in its selectivity for cancer cells. One significant benefit is that PDT offers a less invasive treatment option, which can be particularly appealing to patients. Additionally, PDT can be administered more frequently as needed, thanks to its minimal side effects and the absence of resistance mechanisms that often complicate other treatments. This flexibility allows for better management of the disease. Furthermore, PDT can be effectively combined with other cancer therapies, enhancing overall treatment efficacy while maintaining the advantages of those additional therapies. Another notable aspect of PDT is that it typically results in little to no visible scarring or long-term side effects, which can greatly improve the patient’s quality of life post-treatment. Moreover, PDT allows for quicker treatment courses, making it more convenient for patients who may have busy schedules or other commitments. However, it is crucial to recognize that PDT does have some limitations. Although rare, there can be adverse side effects associated with the therapy, and the effectiveness of PDT can be constrained by the size and exact location of the tumor, which can limit the intensity of the light used (Ma et al., 2024) (Table 2A). Therefore, further research and clinical studies are necessary to fully harness the potential of PDT in cancer treatment.

**TABLE 2:** Advantages and challenges of PDT (Panel A), (Panel B).

N	Advantages	
1	Minimally invasive	PDT employs fiber optics and endoscopes to deliver laser light deep into the body. This method effectively avoids the trauma and pain commonly associated with surgeries like thoracotomy and laparotomy
2	High selectivity	Photosensitizers concentrate in tumor tissues at higher levels than in surrounding normal tissues, and the photodynamic reaction mainly occurs in cancer cells after light exposure, causing minimal damage to normal tissues
3	Low toxicity	Photosensitizers only trigger a phototoxic reaction under specific light exposure conditions, and the parts of the body not exposed to light do not react, having little impact on other organs and tissues
4	Repeatability	Cancer cells do not develop resistance to photosensitizers, and patients can undergo PDT multiple times without increasing toxicity
5	Flexibility	PDT can be used in combination with other treatment methods such as surgery, radiotherapy, and chemotherapy to improve treatment outcomes and help eliminate occult cancer lesions
6	Preservation of organ integrity	For certain tumors, such as laryngeal cancer and skin cancer, PDT can effectively treat cancer while minimizing damage to the structure and function of the affected organ
7	Elimination of occult cancer lesions	PDT can detect and treat tiny cancer nests that are invisible to the naked eye through fluorescence diagnosis, reducing the chance of tumor recurrence
8	Synergistic surgery to improve efficacy	PDT can be used as an adjunct to surgery, reducing tumor burden or eliminating potential lesions to improve surgical success rates and treatment outcomes
9	Activation of anti-tumor immunity	PDT can activate the body’s anti-tumor immune response, providing another means for the body to destroy cancer cells
10	Broad treatment range	PDT is not only applicable to the treatment of various types of cancer but also can be used to treat some non-malignant conditions, such as acne and psoriasis
N.	Challenges	Solutions
1	Tumor selectivity is not high	Conventional photosensitizers may be distributed in both normal and tumor tissues, resulting in low selectivity. To improve selectivity, researchers are developing novel photosensitizers that can be specifically absorbed by tumor cells, for example by targeting molecules to modify photosensitizers to enhance their affinity for tumor tissues
2	Insufficient tissue penetration	Photosensitizers need to have sufficient penetration in biological tissues for light energy activation. Researchers are exploring the use of nanotechnologies, such as gold nanoparticles (AuNPs), to load photosensitizers to enhance the distribution and penetration of photosensitizers in tumor tissues
3	Oxygen dependence	The efficacy of PDT is dependent on the presence of oxygen, and many solid tumors are hypoxic, which limits the efficacy of PDT. To overcome this problem, researchers have developed oxygen-independent photosensitizers, such as oxygen-enhancing photosensitizer nanoparticles that can be activated by low light irradiation, which can increase the amount of oxygen in the tumor site microenvironment to enhance the PDT effect
4	Photostability of photosensitizers	Some photosensitizers may degrade when exposed to light, affecting the effectiveness of treatment. To solve this problem, researchers are developing more stable photosensitizers to improve their stability and efficacy under light exposure
5	Bioavailability of photosensitizers	Water-soluble photosensitizers have limited ability to penetrate biofilms, making it difficult to aggregate in tumor cells. By combining photosensitizers with nanoparticles, its bioavailability and concentration within tumor cells can be increased
6	Phototoxicity of photosensitizers	Photosensitizers may be phototoxic after exposure to light, leading to damage to normal tissues. To reduce this risk, researchers are exploring the use of low-toxicity photosensitizers and reducing the impact on normal tissues by precisely controlling the dose and timing of light
7	Optimization of treatment options	In order to improve the efficacy of PDT, researchers are exploring the use of other treatments in combination with PDT, such as chemotherapy, radiotherapy, immunotherapy, etc., to enhance the treatment effect

Photosensitizers face multiple challenges in their effectiveness against tumors. One significant issue is the insufficient penetration of light into tumor tissue, which hampers the activation of these agents. Additionally, the hypoxic conditions often found in melanoma environments limit the availability of oxygen necessary for type II PDT (Nkune and Abrahamse, 2024; Khaddour et al., 2021). The presence of melanin further complicates matters, as it absorbs light and diminishes the effectiveness of PDT while also stabilizing free radicals. Moreover, melanosomes within melanoma cells provide a protective barrier against anticancer drugs, contributing to the phenomenon of drug resistance (Sharma et al., 2011). Other limitations of PDT include poor solubility, off-target toxicity, and the inherent difficulties associated with treating deep-seated tumors. These challenges have sparked a growing interest in the development of nanotechnology and combination therapies aimed at overcoming these obstacles (Kah et al., 2023).

## 5 Latest research findings on photodynamic therapy

Recent research and PDT methods focus on enhancing specificity and uptake. This is achieved by combining photosensitizers with delivery mechanisms to address existing challenges. In the article by Ma et al. (2024), Photosensitizers are used together with nanoparticle systems and antibodies to improve tumor specificity. The DPTC nano-platform effectively treats hypoxic tumors through PDT and telaprazine (TPZ) reactions, showing significant anti-tumor effects in studies, while MNs enhance targeted accumulation and reduce toxicity, making DPTC-MNs promising for skin tumor treatment.

Research on PDT for skin cancer has significantly advanced the development of new photosensitizers and technologies, particularly in the application of nanomaterials (Chen et al., 2022). Analysis of head and neck cancers indicates that nanomaterials and targeted therapies are key research focuses (Zhan et al., 2022). Nanomaterials are excellent candidates for non-invasive cancer diagnosis and treatment due to their multifunctionality (Li L. et al., 2023). Additionally, nanodrugs designed to target tumor hypoxia have shown promise in PDT, allowing for targeted therapy through hypoxia-responsive drug design, which reduces side effects on normal tissues (Xu et al., 2022). This strategy has demonstrated exceptional effectiveness in chemotherapy, radiotherapy, PDT, and immunotherapy. Graphene oxide nanoparticles show great potential in cancer treatment because of their photosensitivity and biocompatibility. Moreover, chemical modifications can reduce cytotoxicity and improve their effectiveness in both chemotherapy-photothermal therapy and chemotherapy-PDT (Itoo et al., 2022).

The development of new photosensitizers represents a critical focus in PDT research. The use of the iron chelator protoporphyrin IX (PpIX) in photodetection and PDT has demonstrated significant effects. Additionally, iron chelators can enhance PpIX accumulation, thereby improving PDT effectiveness (Magnussen et al., 2022). Furthermore, researchers are exploring the combination of PDT with other treatment methods, including systemic chemotherapy and endoscopic radiofrequency ablation (eRFA), which have shown promising outcomes for extrahepatic bile duct cancer (Möhring et al., 2023). Significant progress has been achieved in applying PDT to glioblastoma treatment, particularly with npe6-mediated PDT, which effectively induces glioblastoma cell death (Kobayashi et al., 2020). Recent advancements in PDT for skin cancer treatment have introduced new photosensitizers and nanomaterials. These innovations enhance treatment efficacy and reduce side effects, thereby offering more options for clinical application.

## 6 The future development trends of photodynamic therapy

PDT has made progress in treating malignant skin tumors but still encounters future challenges and opportunities (Table 2B). We have conducted a thorough evaluation of photodynamic therapy, focusing on its development, potential applications, and how it integrates with new technologies.

### 6.1 Increase treatment depth and reduce side effects

Currently, photodynamic therapy faces a significant limitation: the shallow penetration depth of light, particularly when targeting deep tumors, which results in less effective treatment. To overcome this challenge, researchers are investigating higher energy light sources, like X-rays, to activate photosensitizers and enhance treatment depth (He et al., 2022). Moreover, nanotechnology introduces new possibilities for photodynamic therapy. By combining photosensitizers with nanoparticles, it enhances their accumulation at tumor sites. This approach improves treatment efficacy and reduces harm to surrounding healthy tissues (Wang et al., 2021).

### 6.2 Forecasting the prospects of photodynamic therapy in skin cancer treatment

As technology advances, photodynamic therapy shows great promise for treating skin cancer. New photosensitizers, including polymer and quantum dot types, have been developed. These new agents demonstrate increased photosensitivity and improved biocompatibility (Rejinold et al., 2021). Additionally, combining photodynamic therapy with other therapies like chemotherapy, radiotherapy, and immunotherapy has shown significant synergistic effects that can enhance overall treatment outcomes (Wang et al., 2021).

### 6.3 Analyzing the potential of combining photodynamic therapy with other emerging technologies

Integrating photodynamic therapy with other emerging technologies holds significant promise. For example, combining nanotechnology with immunotherapy can significantly improve the effectiveness of PDT. Nanoparticles can act as carriers for photosensitizers, which helps them accumulate more at tumor sites and delivers immune modulators to strengthen the immune response (Desai et al., 2024). Moreover, combining photodynamic therapy with photothermal therapy shows promising potential. Using photosensitizers alongside photothermal agents can more effectively destroy tumors (Li L. et al., 2023).

PDT combined with chemotherapy (CHT) enhances tumor treatment efficacy. Doxorubicin (DOX) is currently the most commonly used combination chemotherapy drug. Li et al. (2020) used carbon dots (CDs) as nanocarriers to encapsulate the anticancer drug DOX along with 5-ALA. They achieved an encapsulation rate of 83.0%. In this system, CDs serve two roles: acting as a nanoplatform for drug loading and a fluorescent probe for cancer cell diagnosis. Fourier-transform infrared spectroscopy (FTIR) showed that CDs bind to 5-ALA and DOX through different mechanisms. Specifically, they utilize EDC/NHS coupling reactions for 5-ALA and hydrogen bonding for DOX. They also observed that the synergistic effect of laser combined chemotherapy drugs increased the mortality rate of breast cancer MCF-7 cells, and numerous studies have reported that chemotherapy drugs and PSs insert into DNA and disrupt topoisomerase-mediated DNA repair (Babič et al., 2018).

In the future, the advancements in photodynamic therapy for malignant skin tumors will focus on increasing treatment depth, minimizing side effects, creating new types of photosensitizers, and integrating with other technologies. As technology advances, photodynamic therapy is anticipated to play a larger role in treating skin cancer, offering patients more effective treatment options.

## 7 Conclusion

PDT has made significant progress in the treatment of skin cancer, becoming a promising option due to its unique mechanisms and clinical benefits. Successful cases show that PDT is minimally invasive, highly selective, and reproducible in treating superficial skin cancers, including basal cell carcinoma and squamous cell carcinoma. However, PDT faces several clinical challenges, such as adverse reactions to photosensitizers, limited treatment depth, and varying patient responses. Choosing the right photosensitizers and light wavelengths is crucial for effectiveness. Future research should aim to develop new photosensitizers that improve tissue penetration and minimize side effects. Combining nanotechnology with immunotherapy could enhance the efficacy and safety of PDT. An effective combination of PDT with surgery, radiotherapy, and chemotherapy is key to achieving optimal results. For refractory and recurrent skin cancers, the prospects of PDT combined with immunotherapy are promising and warrant further investigation. Despite its great potential for treating skin cancers, PDT’s widespread use must overcome various technical and clinical barriers.

Future research should optimize photosensitizers, increase treatment depth, and explore combination therapies. Through multidisciplinary collaboration and innovation, PDT is expected to play a more significant role in treatment, providing patients with more effective and safer options.

## Abbreviations

PDT, photodynamic therapy; PS, photosensitizer; ROS, reactive oxygen species; VEGF, vascular endothelial growth factor; eRFA, endoscopic radiofrequency ablation; JNK, c-Jun N-terminal kinase; NLRP3, NLR family pyrin domain containing 3; ALA, 5-aminolevulinic acid; NMSC, non-melanoma skin cancer; SCC, squamous cell carcinoma; BCC, basal cell carcinoma; M-PDT, modified 5-aminolevulinic acid photodynamic therapy; N-GSDMD, N-terminal of gasdermin D; PIT, photodynamic immunotherapy; LS-HB, Chlorin e6-C-15-ethyl ester.
